# Cutaneous lichen amyloidosis in multiple endocrine neoplasia type 2A

**DOI:** 10.1210/jcemcr/luag208

**Published:** 2026-08-03

**Authors:** Uzair ul Haq Mir, Mohammad Hayat Bhat, Shahnaz A Mir

**Affiliations:** Department of Endocrinology, Government Medical College, Srinagar 190010, India; Department of Endocrinology, Government Medical College, Srinagar 190010, India; Department of Endocrinology, Government Medical College, Srinagar 190010, India

**Keywords:** multiple endocrine neoplasia type 2A, cutaneous lichen amyloidosis, pheochromocytoma, PHPT, primary hyperparathyroidism

## Image legend

A 50-year-old man presented with a pruritic hyperpigmented rash which had been present on his right scapular region since childhood. A skin biopsy obtained from this scapular lesion stained positive with Congo red and demonstrated amyloid deposits in the papillary dermis, consistent with cutaneous lichen amyloidosis. He also reported recurrent episodes of palpitations, diaphoresis, headache, and severe hypertension along with a previous history of nephrolithiasis. Further workup revealed markedly elevated plasma normetanephrine levels and primary hyperparathyroidism. Computed tomography identified bilateral adrenal masses and parathyroid disease. Genetic testing identified a pathogenic *RET* exon 11 mutation (c.1900T>C; p.Cys634Arg). Despite carrying a high-risk mutation, the patient had not developed medullary thyroid carcinoma. Multiple endocrine neoplasia type 2A (MEN2A) is an autosomal dominant syndrome caused by activating *RET* mutations and is classically characterized by medullary thyroid carcinoma, pheochromocytoma, and primary hyperparathyroidism. Cutaneous lichen amyloidosis is a recognized but uncommon phenotypic manifestation, particularly associated with mutations affecting codon 634 of the *RET* oncogene, and often precedes the development of associated endocrine neoplasia. This case highlights the importance of recognizing cutaneous lichen amyloidosis in unraveling MEN2A [1].

**Figure:**
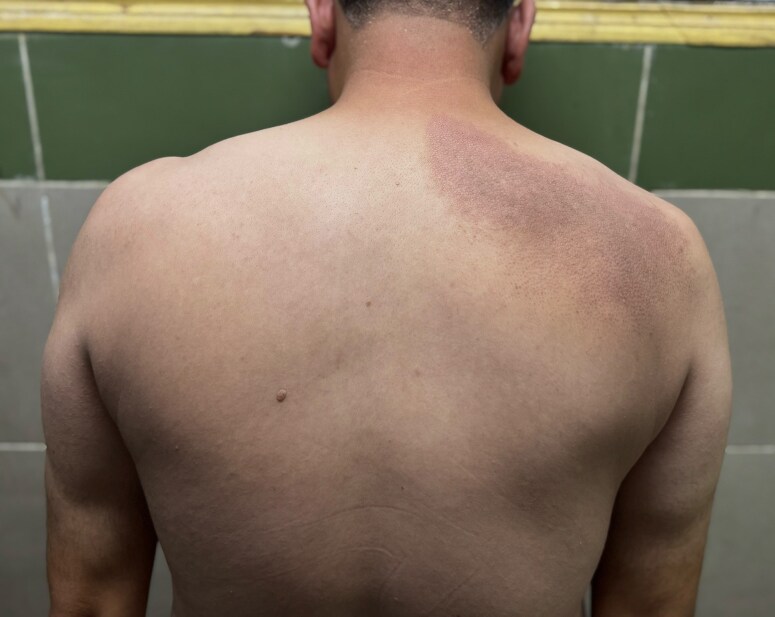


## Contributors

All authors made individual contributions to authorship. U.H.M. and M.H.B. were involved in the clinical diagnosis, workup, and management of the patient. S.A.M. was involved in data collection, literature review, and drafting the initial manuscript. All authors critically reviewed, edited, and approved the final version to be published.

## Data Availability

Original data generated and analyzed during this study are included in this published article.
